# Supplementary material for: Observation of angle-dependent mode conversion and mode hopping in 2D annular antidot lattice
Source: Sci Rep. 2019 Aug 20;9:12138. doi: 10.1038/s41598-019-48565-8 (PMC6702203; doi:10.1038/s41598-019-48565-8)
Supplement: Supplementary file 1 — Dataset 2 [file 41598_2019_48565_MOESM1_ESM.docx]

**Supplementary Material**

**Observation of angle-dependent mode conversion and mode hopping in 2D annular antidot lattice**

Nikita Porwal^1^, Anulekha De^2^, Sucheta Mondal^2^, Koustuv Dutta^2^, Samiran Choudhury^2^, Jaivardhan Sinha^2^, Anjan Barman^2^ and P. K. Datta*^1^

*^1^Department of Physics, Indian Institute of Technology Kharagpur, W.B. 721302, India*

*^2^Department of Condensed Matter Physics and Material Sciences, S. N. Bose National Centre for Basic Sciences, Block JD, Sector III,*

*Salt Lake, Kolkata 700 106, India*

**Correspondence email:* [*pkdatta@phy.iitkgp.ac.in*](mailto:pkdatta@phy.iitkgp.ac.in)

1. **Detailed Simulation to Understand the Origin of Mode Conversion:**

To understand the disappearance of mode 3 of the annular antidot lattice (AAL) with bias field orientation (ϕ), we simulate the SW spectra by gradually varying ϕ at 1° interval, and Fig. S1 shows the simulated spectra for some specific angles. Here, we observe that the frequencies of all three modes periodically vary as described already in the manuscript. The power of these modes shows interesting behaviour for 10°≤ϕ≤30°. Mode 1 is the weakest mode among these three modes. As ϕ increases, power of mode 1 gradually increases up to 30°. Mode 2 has the intermediate power, which further increases up to 20° followed by a gradual decrease up to 30°. Mode 3 is the most intense mode at 10° but it loses power gradually with the increase in ϕ and completely disappears at 22°.


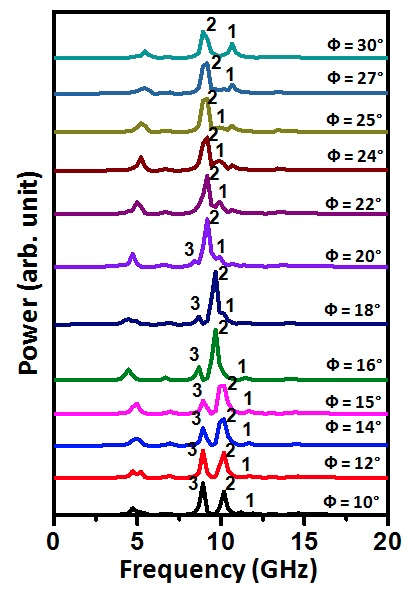


Fig S1: Simulated power vs. frequency spectra of the AAL with the orientation of bias magnetic field (ϕ) from 10° to 30°.

1. **Simulated Mode Frequency as a function of Bias Field Orientation for Dot and Antidot Lattices:**

To understand the origin of the modes of the annular antidot lattice, we simulate constituent dot lattice (DL) and antidot lattice (ADL) of same dimensions. The variation of the simulated SW mode frequencies as a function of ϕ (0°≤ϕ≤ 90°) of the DL is shown in Fig. S2(a) and the ADL is shown in Fig. S2(b). The DL shows two modes. Both of them show four-fold anisotropy with opposite curvature. The theoretical fits to the data give the four-fold anisotropy fields (H_K4_) of 23 Oe and 18 Oe for mode 1 and mode 2, respectively [ref: B. Rana et al. Journal Of Applied Physics 111, 07D503 (2012)]. However, the ADL shows several modes with different behaviours. Mode 1 shows a four-fold anisotropy with H_K4_ = 22 Oe. Mode 2 shows a distorted four-fold symmetry with flattened valley. Mode 3 and mode 5 show a rather complex nature. Its frequency falls sharply from 0° to 20° followed by a gradual increase up to 45° and another gradual decrease from 45° to 70°. From 70° to 90° the frequency increases sharply back to the value observed at 0°. Mode 4 appears and disappears periodically for certain angular range.


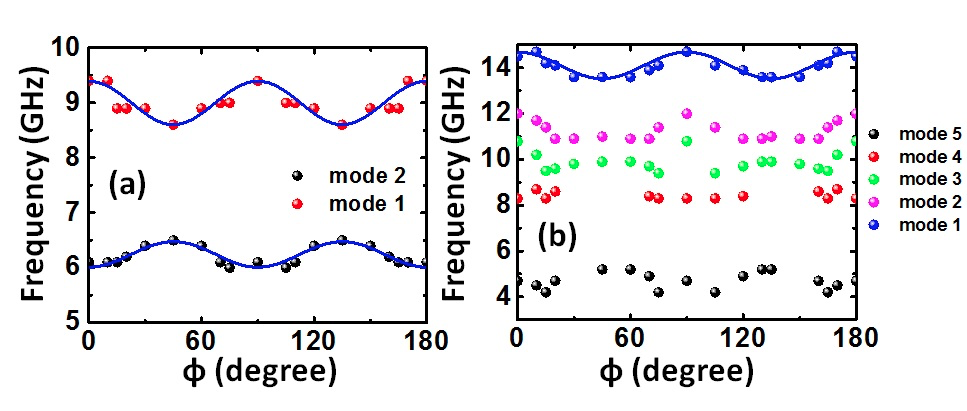


Fig S2: Angular variation of the SW modes with in-plane magnetic field angle obtained from micromagnetic simulations for individual (a) dot lattice and (b) antidot lattice. The solid lines are fit corresponding to the four-fold anisotropy.

1. **Angular Dispersion of Two Lowest Frequency Modes of the Annular Antidot Lattice**

The angular dispersion of the two lowest frequency modes (modes 4 and 5) of the annular antidot lattice (AAL) obtained by experiment and simulation are shown in Fig. S3(a) and (b),


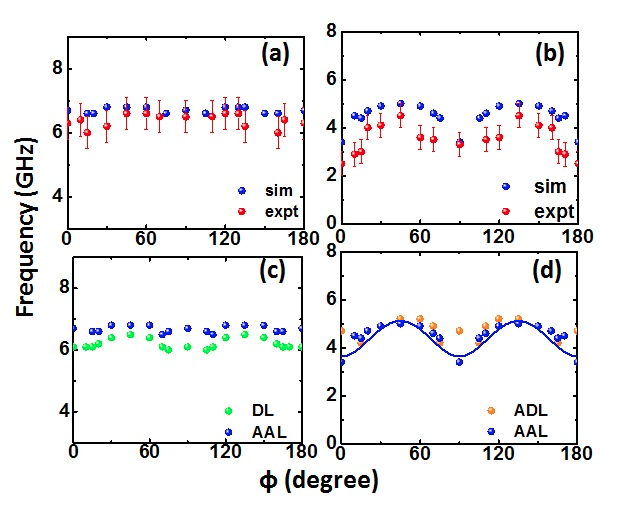


Fig S3: (a) Variation of SW frequency of (a) mode 4 and (b) mode 5 of the annular antidot lattice with the in-plane angle of the bias field varying from 0° to 90° at a fixed H = 1080 Oe obtained from both experiment and simulations. The origin of (c) mode 4 and (d) mode 5 of annular antidot lattice is studied by comparing them with corresponding modes of the constituent DL and ADL. The solid lines are fit corresponding to the four-fold anisotropy.

respectively. We have further compared them with the corresponding modes of the constituent DL and ADL of the same dimensions (Fig.S3(c) and (d)). Mode 4 is found to be mainly contributed by the DL (Fig S3(c)) while mode 5 is mainly contributed by the ADL (Fig S2(d)). Additionally, we also observe that mode 4 of the AAL show distorted four-fold anisotropy and mode 5 shows four-fold anisotropy with H_K4_ = 80 Oe. Also, in the constituent DL (ADL), the mode near 4 GHz (6 GHz) does not exist. However, in the AAL due to strong dipolar interaction between the DL and the ADL, existence of both modes is observed.

**4. Phase profile for mode 4 and mode 5 of the annular antidot lattice**

Figure S4 depicts spatial profiles of mode 4 and 5 of the annular antidot lattice at some specific in-plane bias magnetic field angles, calculated using custom built Matlab code (DotMag). It is found that for mode 4, the main power is mainly concentrated at the edges of the dots (it is contributed by the edge mode of the dots). For mode 5, the main power is mainly concentrated at the edges of the antidots (it is contributed by the edge mode of the antidots).


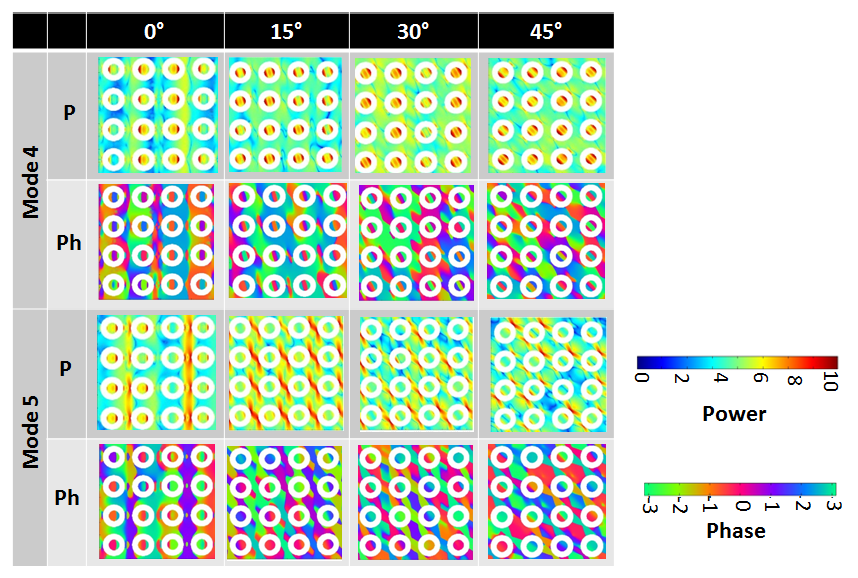


Fig S4. Spin-wave mode profile for mode 4 and 5 of the sample for different orientations of the in-plane bias magnetic field. The corresponding color maps are also shown.

5. **Angular Dispersion of the Annular Antidot Lattice at low bias magnetic field**

Bias field-dependent spin-wave spectra showed some significant variation with a bias magnetic field. To explore this further, we have studied the angular variation of the spin-wave modes at a lower bias field of 620 Oe. The simulated spin-wave spectra at low bias field for some specific angles is shown in Fig. S5(a). In this low bias field also, mode 1 shows distorted four-fold symmetry with flattened valley (Fig. S5(b)). It's frequency initially decreases systematically between 0° ≤ ϕ ≤ 30°, following which it becomes nearly constant (locked) for 30° ≤ ϕ ≤ 60°and then again systematically increases for 60° ≤ ϕ ≤ 90°. Mode 2 (Fig. S5(c)) generally the highest intensity mode for the full angular range. it exhibits mode hopping-like behaviour between two definite frequency levels. Its frequency remains at a constant level for 0° ≤ ϕ ≤ 10°, followed by a sharp reduction to another constant level for 20° ≤ ϕ ≤ 70°, which is repeated periodically. Mode 3 (Fig. S5(d)) appears and disappears periodically at angles of 0° ≤ ϕ ≤ 10° and 10° ≤ ϕ ≤ 80°. However, its variation is in anti-phase to that observed for *H* = 1080 Oe. Mode 4 (Fig. S5(e)) shows mixed four-fold and eight-fold anisotropy. Its frequency initially increases sharply for 0° ≤ ϕ ≤ 30°, then slightly decreases for 0° ≤ ϕ ≤ 45°, again increases for 45° ≤ ϕ ≤ 65°, and finally decreases sharply for 65° ≤ ϕ ≤ 90°. Mode 5 (Fig. S5(f)) shows a complex angular variation and gets merged with the “#” marked mode in some intermediate angles. The “*” marked mode (Fig. S5(g)) exhibits a complex behaviour with a mode-locking in the angular range 25° ≤ ϕ ≤ 65°. For the other angles, it shows a rapid variation with apparently no specific anisotropy. Some higher frequency modes appear in the intermediate angular range, which are not considered in this study.


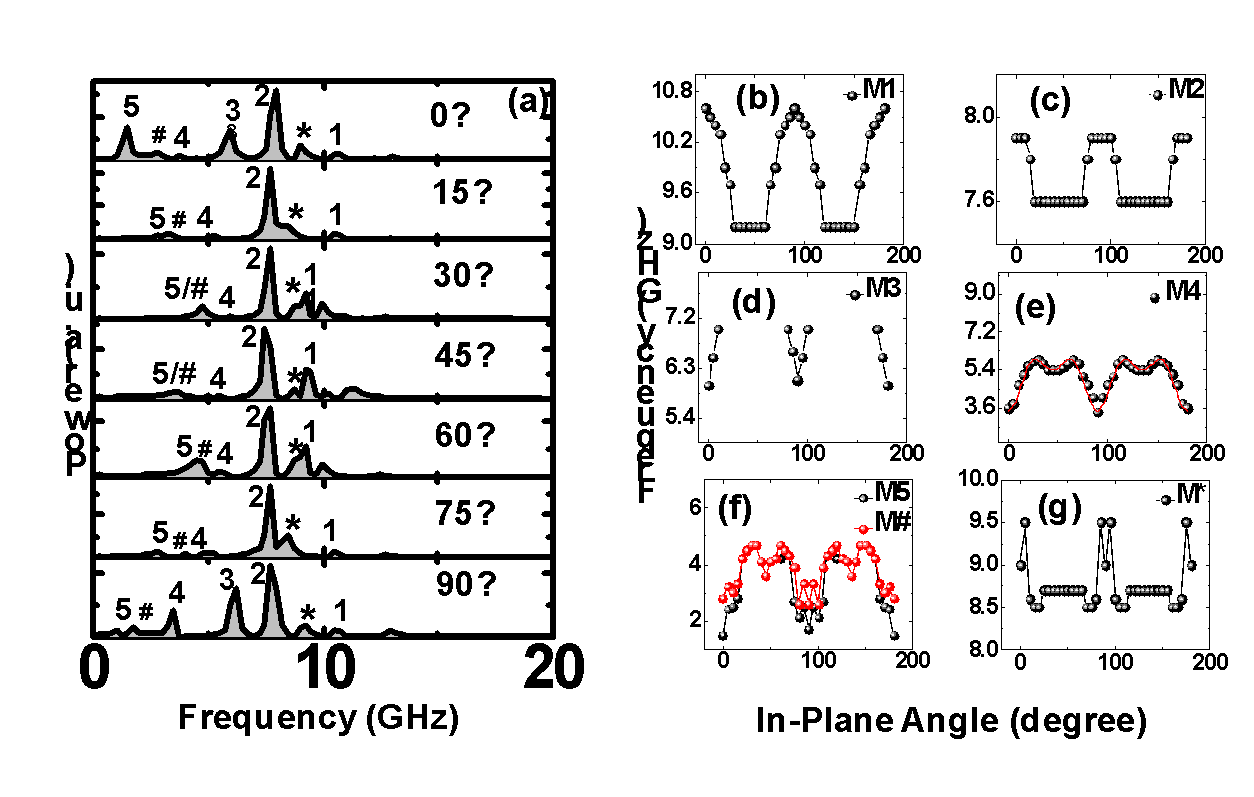


Fig. S5. (a) Simulated SW spectra for some specific angles at low bias field (620 Oe). (b) Angular variation of mode 1, (c) mode 2, (d) mode 3 (e) mode 4 and (f) mode 5 # and (g) mode * at 620 Oe.

**6. Which component is more important in determining spin-wave propagation, dots or antidots?**

The antidots are mainly responsible for the spin-wave propagation/extension, due to the presence of extended channels between the neighboring antidots. The dots are far apart to show strongly collective behaviour to accommodate extended/propagating modes and even their interaction with the antidots does not help in that regard. The dots, however, play some role in modulating the SW propagation/extension within the antidot channel.

To show it more explicitly, we have locally excited the spin-wave dynamics using a sine function containing the frequency of the resonant mode at the region shown in Fig. S6 by the grey bar of 2380 × 60 nm^2^ area. We observe that the SW corresponding to mode 1 propagates to a large distance along the vertical direction with significant power at ϕ = 0° in ADL. With increasing ϕ, the propagation gradually decreases and stops propagating at 45° in the ADL. However, in the dots, the spin-waves are confined and do not propagate due to large interdot distance between the dots.

*
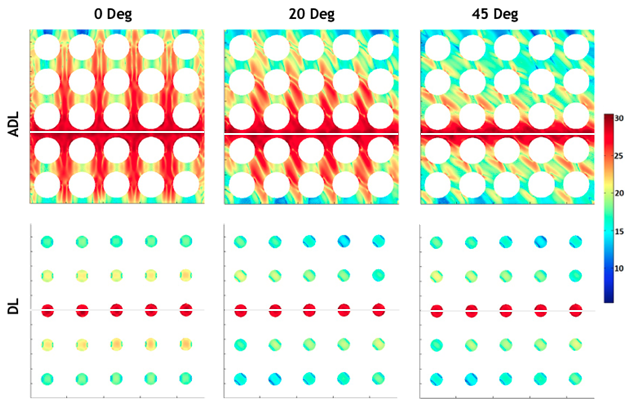
*

Fig S6: Simulated power profiles of spin-wave mode 1 excited locally over the rectangular strip in the ADL and DL for ϕ = 0°, ϕ = 20° and ϕ = 45° geometries.
